# *Mycobacterium fortuitum* Infection Mimicking an Odontogenic Abscess: Report of a Pediatric Case with Relevant Literature Analysis

**DOI:** 10.3390/pathogens14060513

**Published:** 2025-05-22

**Authors:** Giuseppe Barile, Luisa Limongelli, Marta Forte, Tommaso Corsalini, Saverio Capodiferro, Massimo Corsalini

**Affiliations:** Department of Interdisciplinary Medicine, University of Bari “Aldo Moro”, Piazza Giulio Cesare, 11, 70124 Bari, Italy; luisa.limongelli@uniba.it (L.L.); tommasocorsalini@gmail.com (T.C.); saverio.capodiferro@uniba.it (S.C.); massimo.corsalini@uniba.it (M.C.)

**Keywords:** dental abscess, dental infection, endodontics, fine-needle aspiration, pediatric dentistry

## Abstract

Non-tuberculous mycobacteria (NTM) are saprophytes of both soil and water that may cause infection with a high risk of dissemination, mainly in immunocompromised patients. Most NTM infections occur in the lungs, while uncommon localizations are the skin, soft tissues, musculoskeletal apparatus, and lymphatic system. The possible relationship between NTM infections and dental procedures is still unclear. The authors reported a rare manifestation of NTM infection occurring in a 6-year-old girl who developed sub-mandibular swelling related to a necrotic tooth, thus mimicking an abscess of odontogenic origin. Fine-needle aspiration biopsy of the sub-mandibular swelling and the following microbiologic investigation showed infection sustained by the *Mycobacterium fortuitum* complex. After the medical and surgical treatment, the patient completely recovered after 8 months. A review of the relevant literature was carried out to deepen the clinical and microbiological aspects of such a rare occurrence.

## 1. Introduction

As bacterial and viral infections, mycobacterial infections can occur both in soft and hard head and neck tissues, conventionally distinguished as the Mycobacterium Tuberculosis complex (TM) and Non-Tuberculous Mycobacteria (NTM) starting from the study by Runyon in 1959 [1]. In contrast with TM, NTM are environmentally saprophytic and non-pathogenic mycobacteria. When virulent, they mainly affect the lung (approximately 90% of cases) or manifest as lymphadenitis and soft/hard tissue suppurative infections almost exclusively in immunocompromised patients [2]. Few studies worldwide have reported NTM infections after dental treatments in pediatric patients [3,4,5]. It appears as sub-mandibular swelling, which does not respond to conventional therapy, such as root canal treatment and amoxicillin systemic antibiotic therapy. However, differential diagnosis of submandibular swelling is always challenging for general dental practitioners who do not have experience in the field. The main factor responsible for NTM entry through a decayed/necrotic tooth could be direct soil contamination or the use of contaminated dental unit waterlines during dental procedures [6]. If not treated with the right timing, NTM infections in children could lead to critical and severe complications that could also require intensive care [7].

This paper reports a challenging differential diagnosis of an NTM infection during a common odontogenic infection and its problematic management. The relevant literature was analyzed to deepen the rare incidence rate, its diagnosis, and the proposed treatment.

## 2. Case Presentation

A 6-year-old female patient was brought to our attention for rapidly growing left submandibular swelling associated with flushed skin without fever and no local multiple lymph node involvement. One month before, the patient was first subjected to composite resin restoration of a decayed lower second deciduous molar (7.5). Ten days after the restoration, the patient reported pain and the appearance of a gingival fistula, so her general dental practitioner performed root canal treatment of the necrotic tooth without administering antibiotic therapy. After seven days, the ipsilateral submandibular swelling occurred. An oral antibiotic therapy (875 mg of amoxicillin with 125 mg of clavulanate twice per day for 7 days) was set without evident resolution, so the patient was quickly sent to the Policlinic Hospital of Bari for further investigations. A submandibular ultrasonography (US) was performed, showing an area of colliquating lymphoglandular tissue associated with chronic inflammation (approximately 39 mm × 23 mm × 28 mm), and the orthopantomography (OPG) was provided by their dentist. According to their parents, the clinicians decided to extract the involved tooth.

Nevertheless, the submandibular swelling grew progressively in the following days. The patient was conscious but very scared and poorly compliant. Their body temperature was 36.4, and their blood test was as follows: albumine at 4.3 g/dL, total bilirubin at 0.8 mg/dL, CRP at 35.8 mg/dL, HgB at 13.7 g/dL, RBC at 4.7 mill/mm^3^, and WBC at 12.7 with a neutrophil predominance (21.2%), which does not indicate a severe infection. A US-guided fine-needle aspiration (FNA) was performed to collect material for cytological and histological examination as well as microbiological and antimicrobial susceptibility tests (antibiogram), while antibiotic therapy was changed (1 gr of ceftriaxone per day i.v.). The preliminary results showed a possible mycobacterial-induced necrotizing lymph adenomegaly, but the following Mantoux skin test was negative. Under conscious sedation by nitrous oxide and oxygen, the swelling incision and oral fistula debridement with removal of the necrotic tissue with adjunctive histological examination was performed. An iodoform gauze was positioned in the submandibular extraoral wound to allow for drainage, and it was repeatedly changed for the following three weeks without discoloration of the skin or allergic reactions. No surgical complications were registered, with no facial nerve branch weakness and no superinfection of the suture. The histological examination showed dense granulation tissue, areas of necrosis, non-specific inflammatory infiltrates, polymorphonuclear leucocytes, and histiocytes, allowing for a definitive diagnosis of necrotizing granulomatous inflammation. The microbiological characterization (GenoType Mycobacterium CM/AS, Hain Lifescience, Nehren, Germany) found an NTM infection by the *Mycobacterium fortuitum* complex. The antibiogram showed a sensitivity of the *Mycobacterium fortuitum* complex against macrolides, so the patient received 500 mg of oral clarithromycin twice daily, one week every month, for five months. Complete healing without retractive scars was observed three months after the complete antibiotic therapy. The follow-up showed the absence of any systemic and local complications at 1 year.

## 3. Clinical Case

**Figure 1:**
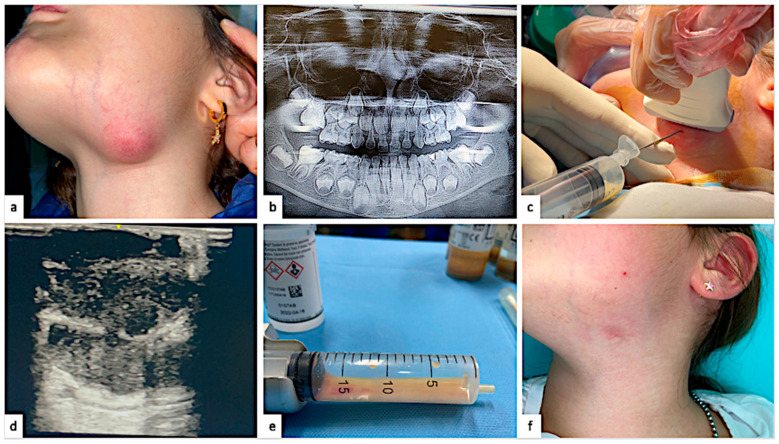
(**a**) The submandibular abscess occurred 8 days after the endodontic treatment without root canal filling. (**b**) Orthopantomography shows a deep mesial cavity on 7.5, associated with periapical radiolucency. (**c**) Fine-needle aspiration biopsy guided by ultrasonography. (**d**) Ultrasonography imaging shows a wide, non-homogeneous structure containing a central area of colliquative necrosis. The histological examination showed dense granulation tissue, areas of necrosis, non-specific inflammatory infiltrates, polymorphonuclear leucocytes, and histiocytes, allowing for a definitive diagnosis of necrotizing granulomatous inflammation. (**e**) Fine-needle aspiration sample. The microbiological characterization found an NTM infection by the *Mycobacterium fortuitum* complex. (**f**) Complete healing occurred after 8 months without scars.

The figure below summarizes the clinical case (Figure 1).

## 4. Discussion

*Mycobacterium tuberculosis* and *Mycobacterium leprae* are responsible for Tuberculosis and leprosy and are historically recognized as the oldest infective diseases, also found in skeletons dated 4000 years [8]. Leprosy is near eradication, whereas Tuberculosis is the second most fatal infection after COVID-19 nowadays, with a downward trending incidence but killing 1.8 million people worldwide in 2021 and causing 10 million cases per year [9]. Indeed, Asia and Africa remain the countries with the highest incidence, where Tuberculosis is the first cause of death in patients with AIDS and HIV [8,9]. In contrast, NTM infections show lower incidence and prevalence than TM in industrialized countries (150.000 cases of NTM per year) [10].

The NTM disease group comprises over 200 species of mycobacteria classifiable according to pigmentation, clinical manifestation, and growth speed (Runyon classification) in slow-growing NTM and rapid-growing NTM [11]; NTM are widespread in soil, drinking water, ice water, swimming pools, and the air and cause pulmonary and extrapulmonary opportunistic infections [12]. Pulmonary infections represent 90% of cases and develop a Tuberculosis-like fibro-cavitary or multifocal nodular bronchiectasis form, while extrapulmonary infections occur in the skin and soft tissues by superinfection of traumatic, surgical, or cosmetic wounds in a contaminated environment [13]. Rapid-growing NTM species such as *Mycobacterium abscessus* and *Mycobacterium fortuitum* frequently cause skin infections such as papules, pustules, abscesses, or ulcerations [14]. Tenosynovitis, vertebral osteomyelitis, and prosthetic joint infections may occur when the musculoskeletal apparatus is targeted. The *Mycobacterium fortuitum* complex is a non-pigmented rapid-growth NTM that colonizes water-based solutions and is responsible for healthcare-associated infections, skin, and soft tissue abscesses due to cosmetic surgery procedures, lower extremity furunculosis in immunocompetent adults, and cervical lymphadenitis in immunocompetent children [15].

In this case, the NTM infection was coincidental with an odontogenic infection, so the etiopathogenesis is challenging to understand as socio-economic factors and pre-existing systemic diseases have been excluded. If the NTM infection occurs during an odontogenic infection, the clinician must consider two possible causes that allow NTM entry, namely the superinfection of an endodontically treated tooth or a coincidental primary infection. *M. fortuitum* is usually found in water and soil, and oral contamination is common among children because of their hand-to-mouth behaviors [16].

Moreover, the NTM entry after dental procedures could be related to contaminated dental unit waterlines (DUWs). In fact, DUWs could be considered a reservoir of many bacteria. When the water flow becomes stagnant, a microbiological biofilm could colonize the inner pipes, which may lead to external contamination. The American Dental Association (ADA) suggests a contamination level of <200 colony-forming units (CFUs)/mL^−1^ [17].

In our case, the mycobacteria could make their way through the tooth, as well as the hard and soft tissue, causing osteomyelitis and purulent lymphadenopathy, nearly as the TBC [18], and the neck infection followed timely dental procedures for the treatment of a necrotic tooth, so establishing the exact cause of the descripted infection is not possible.

The prevention of NTM infections could play a pivotal role in reducing the rate of such occurrences. Prevention strategies must follow two methods: in the first one, each child must be adequately educated by their parents to minimize hand-to-mouth behaviors, which permit the first entrance of NTM into the host’s body. The second one involves the systematic control of healthcare facilities, with a rigid disinfection protocol of waterlines [19]. The literature strongly suggests continuing water quality monitoring, using high-level disinfectants and point-of-use water filters, having DUWs be constantly chlorinated, having every handpiece equipped with anti-retraction valves, and having no dead ends in plumbing, without neglecting the educational behaviors from parents [20,21,22].

After the prevention strategies, a rapid diagnosis is required to reduce the disease’s course and morbidity. Histological examination and microbiological culture are mandatory for diagnosing NTM infection, and those steps require from a few days to several weeks. Although the literature supports open biopsy in NTM infections, FNA could be preferred for its minimal invasiveness and high accuracy [23], especially when monitored by US [24]. Moreover, whole-genome sequencing (WGS) is considered the gold standard technique that allows for the characterization of the isolated NTM [25]. WGS combined with real-time PCR could reach a high sensitivity level of diagnostics, preventing late therapy and worsening the outcome [26].

The therapeutic options are surgical and non-surgical treatment [27]. The surgical therapy of pediatric NTM-induced lymphadenitis depends on the localization of the infection, considering its proximity to the facial nerve. When possible, the complete excision of the lesion is preferred. On the other hand, incision and superficial debridement are considered more conservative approaches that can reduce facial branch issues [28]. The non-surgical treatment consists of a systemic antibiotic therapy, which could be different on a case-by-case basis. The NTM are often sensitive to macrolides, which could be used alone or combined with rifampin or ethambutol, depending on the infectious disease specialist involved [29]. However, the antibiotic regimen could be variable across different institutions, so the antibiogram is invalid. In the presented case, we preferred to carry out a combined surgical and medical therapy, which essentially consists of necrotic lymph node removal and superficial debridement of the granulation tissue. This was performed by a plastic surgeon with many years of experience in the field, and the therapy was associated with macrolide therapy, suggested by the NTM-specific sensitivity shown in the antibiogram.

The complete healing evaluation must follow specific clinical criteria, which are presented as follows [30]:

- The duration of the treatment: A fast treatment can reduce the overall morbidity of the disease and the percentage of complications.
- The absence of facial nerve branch issues, which is the worst complication after the surgery.
- The absence of a retractive scar, which could affect the facial esthetic of the patient, especially a pediatric one.
- The restitutio ad integrum of the affected site.

Diagnostic and surgical protocols are illustrated in the IPOG guidelines, which describe the workflow the clinicians must follow in these cases [30]. After the medical and surgical therapy, the healing time of our patient was completed in three months, considering the criteria discussed below, which is consistent with data reported in the literature, which generally ranges between five and eight months [14,27].

We focused on the current literature to deepen our understanding of the rare incidence of an NTM infection during an odontogenic infection. The few studies found are reported below.

Pérez-Alfonzo et al. [3] reported three odontogenic sinus tract infection cases due to NTM associated with dental pathological conditions or following dental procedures with painless, rapidly growing swelling in the submandibular and lateral–cervical regions. Two patients developed extraoral sinus tracts, respectively, four and two months after wisdom tooth extractions, while the remaining developed after an inadequate root canal treatment.

After this report, Castellano Realpe et al. decided to evaluate the presence of NTM in Venezuela’s DUW, finding a rate of 56% of DUWs contaminated by NTM in Caracas [31].

An interesting study by Singh et al. reported the most significant outbreak registered, in which 22 children showed a confirmed NTM infection by *Mycobacterium abscessus* in a sample of 1082 pediatric patients who underwent dental pulpotomy [4], concluding that NTM infection could represent a complication in dental procedures and suggesting the use of sterile water during them.

Peralta et al. found a mycobacterial count above the level recommended by the American Dental Association in water samples from dental units of a private dental clinic, thus explaining the outbreak of 20 children affected by *Mycobacterium abscessus* after a dental pulpotomy [21].

A similar outbreak was reported in an American community, with 14 cases of children who showed cervical lymphadenitis after dental pulpotomy [22]. They were affected by *M. abscessus* and treated with amikacin antibiotic therapy and debridement surgical therapy. Complete recovery occurred after 6 months.

Zhukhovitskaya et al. reported that *Mycobacterium abscessus* is the most common cause of neck lymphadenopathy in children after dental pulpotomy [5], explaining the lack of direct correlation between NTM infections and specific dental procedures for the NTM found also in healthy people. According to them, another outbreak of 24 children in 2 years was affected by *M. ascessus* neck infection after dental pulpotomy in the USA [22]. All the relevant literature on pediatric NTM infection after dental procedures is summarized in the table below (Table 1).

Considering such data, the case presented above is the second case of *M. fortuitum* lymphadenopathy in a patient following a dental procedure. Hence, it needs to be presented for its sporadic presentation. In these cases, the differential diagnosis with a common odontogenic infection is very challenging, especially for an inexperienced dental clinician. So, we suggest that the diagnosis and treatment must be conducted by experienced and trained clinicians and surgeons, not by a private practitioner, who has the pivotal role of quickly understanding this rare pathology and sending the patient to a specialized hospital, reducing diagnostic delay. In fact, the above presented case involved a general dentist, a pediatric dentist, an oral surgeon, anesthesiologists, radiologists, a plastic surgeon, a pathologist, and microbiologists to reach the best outcome. The importance of a trained and experienced multidisciplinary team must be stressed in such complex cases, which could be resolved if the treatment follows precise guidelines [30].

The main limitation of the current case report is the lack of water samples from the dental unit of the patient’s GDP to measure the eventual concentration of NTM, so establishing a direct relationship is not possible. The lack of histopathologic examination images in detail regarding the methodology used for microbiological identification and susceptibility testing could reduce the diagnostic validity of this case report and their educational impact. Moreover, no additional information about managing pediatric NTM infection was added, as the international guidelines were followed.

## 5. Conclusions

NTM infections during dental treatment in pediatric patients could be considered a rare occurrence, but general dental practitioners must be aware of them to reduce diagnostic delay. A diagnostic delay could lead to severe local and systemic complications. The authors strongly suggest preventive strategies and an experienced multidisciplinary team to determine the correct diagnosis and treatment.

## Figures and Tables

**Table 1:** Children’s NTM infections after dental procedures.

Authors	Year and Country	Number of Patients	Mean Age	NTM	Healing Mean Time
Perez-Alfonso et al. [3]	2020, Venezuela	3	17 years	*M. fortuitum* *M. abscessus* *M. peregrinum*	5 months
Singh et al. [4]	2016, California, USA	22	6 years	19 *M. abscessus* 1 *M. chelonae* 2 NTM not further specified	1 year
Peralta et al. [21]	2015, Georgia	20	7 years	*M. abscessus*	4 months
Hatzenbuehler [22]	2015, USA	14	7 years	*M. abscessus*	6 months

## Data Availability

The original contributions presented in this study are included in the article. Further inquiries can be directed to the corresponding authors.
